# Body Shaming and Women's Mental Health: Public Health Challenge for Gender Equity—A Perspective Article

**DOI:** 10.1002/hsr2.72604

**Published:** 2026-06-28

**Authors:** Shani Kondo Omari, Lydia Issaria Mosses, Hussein Hassan Mwanjali

**Affiliations:** ^1^ Tanzania Inclusion and Development Forum (TIDF) Dar es Salaam Tanzania

**Keywords:** body image, body shaming, gender equity, public health, social media, stigma cultural norms, women's mental health

## Abstract

**Background and Aims:**

Body shaming refers to the stigmatization of individuals based on body weight, shape, or appearance; it is an under‐recognized determinant of women's mental health. It contributes to low self‐esteem, depression, anxiety, and diminished quality of life, while discouraging health‐seeking behaviors and reinforcing gender inequities. Despite growing global recognition of body shaming as a public health concern, evidence on its prevalence, cultural dynamics, and impact in African contexts remains limited. This review aims to synthesize current evidence on body shaming, examine existing interventions, and identify gaps particularly within African settings.

**Methods:**

A narrative review of published literature, global health frameworks, and advocacy reports was conducted. Sources included guidance from the World Health Organization (WHO) and the American Psychological Association (APA), evidence‐based therapeutic interventions, digital advocacy movements, and policy frameworks such as the African Union's Agenda 2063.

**Results:**

The WHO and APA now formally recognize appearance‐related distress as a mental health determinant. Evidence‐based interventions including Cognitive Behavioral Therapy (CBT), Compassion‐Focused Therapy (CFT), and school‐based programs such as the Body Project demonstrate measurable reductions in body dissatisfaction and associated psychological risks. Large‐scale initiatives like the Dove Self‐Esteem Project have reached millions of young women globally, while digital movements (#BodyPositivity, #DetoxYourFeed) and mobile mental health platforms continue to expand access and awareness. However, challenges persist: deeply rooted cultural norms that privilege specific body types, limited African‐focused research, inadequate mental health infrastructure, the paradoxical role of social media, and weak policy enforcement.

**Conclusion:**

Addressing body shaming requires intersectional, culturally sensitive research, integration of screening and interventions within healthcare systems, strengthened digital literacy, and cross‐sectoral policy action. Community‐driven campaigns and women's empowerment are central to challenging stigma and celebrating diversity. Systemic change across healthcare, education, media, and policy is essential to promote healthier self‐perceptions, enhance mental wellbeing, and advance gender equity.

## 1 Introduction

Body shaming refers to the negative evaluation, criticism, or stigmatization of individuals based on aspects of their physical appearance such as body weight, shape, skin tone, age‐related features, or other visible characteristics [1]. It constitutes a form of appearance‐based stigma that undermines self‐esteem, reinforces social hierarchies, and contributes to profound psychological distress. Beyond individual harm, body shaming perpetuates structural inequalities by reinforcing exclusionary beauty norms and regulating women's bodies as a site of social control [1, 2].

The proliferation of social media platforms, alongside deeply entrenched cultural norms and globalized beauty standards, has amplified exposure to unrealistic ideals of femininity and attractiveness. Constant comparison with curated images perpetuates insecurities and normalizes derogatory commentary on women's appearances [3]. In many contexts, cultural practices further valorize certain body types, skin shades, or hair textures, intensifying pressures to conform and reinforcing systemic inequalities [4]. These dynamics reinforce internalized shame and social discrimination [5]. Women face higher rates of eating disorders, depression, and anxiety, with body shaming also reducing health‐seeking behaviors and overall quality of life [2, 6, 7]. In Nigeria, university students experiencing body shaming also show reduced academic engagement and psychosocial stress, illustrating the broader social consequences of appearance‐based stigma [8].

Two theoretical frameworks underpin the conceptual pathways explored in this article. Social Comparison Theory holds that individuals assess their own attributes by comparing themselves to others, especially where no objective standard exists [9]. Exposure to idealized body images through peer interaction, family commentary, or algorithmically curated social media content drives upward social comparisons that erode self‐evaluation and deepen body dissatisfaction [3, 4]. Objectification Theory offers a complementary lens, arguing that women are socialized to internalize an observer's perspective on their own bodies. This process of self‐objectification generates habitual body monitoring, shame, and appearance‐related anxiety, which in turn heighten vulnerability to depression, disordered eating, and diminished psychological wellbeing [10]. Together, these frameworks illuminate the internal psychological processes through which social and cultural exposure to body shaming produces measurable harm and explain why its consequences extend beyond individual distress into structural inequity.

The purpose of this article is to critically examine advances, challenges, and future directions in addressing body shaming among women, with a focus on global perspectives and their implications for Africa. It highlights the urgent need for integrated interventions that combine policy, education, mental health services, and digital regulation to dismantle harmful beauty norms and promote inclusive representations of women's bodies. Ultimately, reframing body shaming as a central determinant of women's mental health and social equity is essential if public health and gender justice agendas are to be advanced.

**Figure 1:**
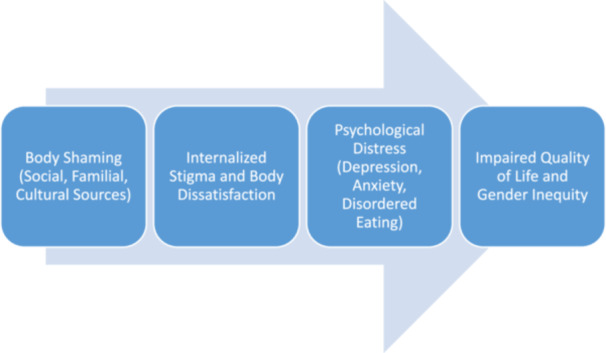
Four‐stage conceptual pathway of body shaming and women's mental health outcomes. The model shows how body shaming from social, familial, and cultural sources, peer commentary, family messaging, culturally valorized beauty norms leads to internalized self‐objectification with body dissatisfaction, shame, and low self‐esteem. This escalates into clinical psychological distress including depression, anxiety, disordered eating, and social withdrawal. The final stage demonstrates consequences: impaired quality of life, reduced health‐seeking behaviors, and entrenchment of gender inequities. The pathway is grounded in Social Comparison Theory and Self‐Objectification Theory.

**Figure 2:**
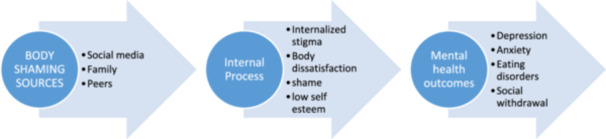
Internal psychological mechanisms linking body shaming exposure to clinical mental health outcomes. Repeated exposure to body shaming through social media, family, and peers triggers upward social comparisons and internalized stigma. This generates body dissatisfaction, shame, and low self‐esteem, the actual psychological mechanisms through which external appearance‐based stigma becomes clinical harm. These states lead into depression, anxiety, eating disorders, and social withdrawal. Feedback loops show how psychological distress reinforces body dissatisfaction and shame in cycles. The figure explains why cognitive and compassion‐focused interventions work: they target self‐comparison processes, reduce shame, and build self‐esteem, interrupting the pathway from exposure to adverse mental health outcomes.

Given the complex and multilevel nature of body shaming, a clear conceptual understanding of its pathways is essential. Figures 1 and 2 illustrate how exposure to appearance‐based stigma from social, familial, peer, and cultural sources may initiate internal psychological processes that contribute to adverse mental health outcomes and broader social consequences for women.

## 2 Current Advances in Addressing Body Shaming in Women

### 2.1 Global Recognition of Body Shaming as a Public Health Issue

In recent years, body shaming that was once dismissed as a matter of personal insecurity has now gained critical recognition as a significant public health issue. Both the World Health Organization (WHO) and the American Psychological Association (APA) now frame body image disturbance as a determinant of mental health, with implications for the prevention of depression, anxiety, and eating disorders [7]. The International Classification of Diseases (ICD‐11) has also acknowledged the burden of appearance‐related distress by including body dysmorphic disorder and weight‐related stigma in its mental and behavioral disorders category, signaling global medical recognition of its impact [11]. Evidence consistently shows that body shaming is strongly linked to disordered eating, low self‐esteem, depression, and social withdrawal, particularly among adolescent girls and women [12].

Global advocacy campaigns have further accelerated recognition of the issue. For instance, the Dove Self‐Esteem Project, launched in 2004, has become one of the largest worldwide initiatives promoting body confidence through evidence‐based educational programs [13]. To date, it has reached more than 82 million young people across 150 countries, with a stated goal of empowering 250 million youth by 2030 [14]. Research from Dove underscores the pervasive nature of appearance‐related anxiety with nearly 80% of girls globally report opting out of essential life activities including sports, school attendance, or engaging with peers because of body image concerns [14]. This shift in framing which is supported by both international health authorities and large‐scale campaigns marks body shaming as no longer trivialized, but instead recognized as a key driver of global mental health disparities.

### 2.2 Impact of Social Media and Digital Advocacy Movements

The rise of social media has both intensified body image pressures and created platforms for counter‐narratives promoting body acceptance. Digital platforms are architecturally designed to maximize engagement, and their recommendation algorithms achieve this by prioritizing aspirational and idealized content, contents that generates emotional reaction, including appearance‐based comparison and insecurity [15]. Platforms such as Instagram and TikTok amplify thin‐ideal content to users who engage with it even briefly, creating a self‐reinforcing loop in which the algorithm interprets engagement as preference and delivers progressively more appearance‐comparison material [3, 16]. A single interaction with body‐focused content can therefore trigger an escalating feed of idealized imagery that is difficult to exit without deliberate action—and it is precisely this repeated, high‐frequency algorithmic exposure, rather than occasional social media use, that drives internalized body dissatisfaction and increases vulnerability to depression and disordered eating [2, 3].

What makes this harm particularly difficult to address is its invisibility. Unlike an identifiable act of body shaming, algorithmic content delivery operates silently, shaping a user's feed through prior engagement patterns without their awareness [3, 15]. This invisibility is compounded by scale—algorithms operate continuously across every session, producing a cumulative exposure burden that no single piece of content would generate alone. The gendered dimension of this process also warrants attention: female‐presenting accounts consistently receive disproportionately higher volumes of appearance‐related content than male‐presenting accounts, reflecting the commercial logic of beauty and diet industries that advertise heavily on these platforms [15, 16]. In African contexts, algorithmically delivered Western beauty ideals intersect with existing local appearance norms around body size, skin tone, and femininity, making their psychological impact additive rather than independent [3, 17]. In response, global advocacy campaigns have emerged to counter these pressures. Hashtag movements such as #BodyPositivity and #EffYourBeautyStandards, alongside Dove's campaigns like #DetoxYourFeed and #NoDigitalDistortion, aim to foster inclusivity and critical engagement with media content [13]. TikTok has also introduced body positive content guidelines, banning harmful diet‐related material to protect vulnerable users [16]. Influencers, NGOs, and media campaigns collectively contribute to reshaping online spaces, emphasizing self‐acceptance and diversity. These efforts demonstrate growing recognition that digital environments must be intentionally structured to support mental wellbeing rather than undermine it.

### 2.3 Evidence‐Based Psychological and Clinical Interventions

Promising clinical and psychological strategies have emerged to mitigate the mental health consequences of body shaming [18]. Evidence also shows that parenting processes and self‐esteem play a key role in social adjustment among students with negative body image, highlighting the importance of family‐ and community‐inclusive interventions [19]. Cognitive Behavioral Therapy (CBT) remains a cornerstone intervention, addressing distorted thought patterns and negative self‐perception associated with eating disorders and body dissatisfaction [20]. Similarly, Compassion‐Focused Therapy (CFT) has shown effectiveness in fostering self‐acceptance and reducing shame‐related distress [21].

School‐based prevention programs targeting media literacy, self‐esteem, and body image resilience have also demonstrated substantial benefits. Programs such as New Moves integrate physical education with education on healthy body image and eating behaviors, highlighting the value of embedding interventions within community and educational systems [22]. The Body Project Collaborative, a Stanford‐ and WHO‐supported cognitive‐dissonance‐based prevention program, has been widely implemented across culturally diverse settings. A meta‐analysis of 56 studies reported moderate reductions in thin‐ideal internalization, body dissatisfaction, and eating disorder risk [18]. Globally, the Body Project reaches approximately 3.5 million girls in 125 countries, demonstrating scalability and adaptability [23].

Innovations in digital mental health interventions are also expanding access. Mobile platforms such as SilverCloud and Woebot provide interactive, evidence‐based programs for body image improvement and early intervention, particularly in populations with limited access to in‐person care [24].

The scalability of these interventions in rural African contexts, however, requires honest consideration. The psychiatrist‐to‐population ratio across sub‐Saharan Africa remains among the lowest globally estimated at fewer than 1 per 100,000 people in many countries making specialist‐delivered CBT or CFT structurally inaccessible for the majority of affected women [25, 26]. Task‐shifting offers a practical and evidence‐supported pathway. In this model, abbreviated and structured versions of CBT and CFT protocols are delivered not by clinical psychologists but by trained lay health workers, community health volunteers, teachers, or peer educators who receive supervised training in core therapeutic techniques [25]. Evidence from Africa demonstrates that lay health workers can deliver psychosocial interventions including psychoeducation on self‐esteem and shame with outcomes comparable to specialist delivery when supervision structures are maintained [25, 27]. The critical condition is that task‐shifting is not simply a matter of handing a clinical manual to an untrained person; it requires adapted materials, ongoing supervision, and fidelity monitoring.

Cultural adaptation is equally non‐negotiable and goes beyond translation. In many African settings, body dissatisfaction does not present through the same cultural scripts that CBT was originally designed to address, thin‐ideal internalization rooted in Western media exposure may coexist with, or be secondary to, community‐level shaming tied to fertility expectations, skin tone, or postpartum body changes [17, 28]. Effective cultural adaptation therefore involves, at minimum, replacing illustrative examples and case vignettes with locally recognizable scenarios; engaging community and religious leaders in framing body acceptance within culturally resonant values rather than importing externally derived self‐esteem language; and piloting adapted materials with target communities before scale‐up [25]. In South Africa, task‐shifted psychosocial programs incorporating local idioms of distress and community‐based group formats have demonstrated feasibility and acceptability [27]. In Nigeria, school‐based programs that embedded media literacy within existing health education classes rather than introducing standalone curricula showed stronger uptake among urban youth [17]. These examples suggest that the architecture of delivery matters as much as the clinical content. While these interventions are evidence‐based, their implementation in low‐ and middle‐income countries faces challenges including limited trained mental health professionals, high costs, and cultural differences in body image norms [25]. School‐ or community‐based delivery models, task‐shifting to trained teachers or peer educators, and mobile health platforms may provide cost‐effective alternatives [23, 27, 29, 30, 31, 32]. However, interventions must be culturally adapted, consider local digital access and literacy, and be tested in context‐specific research to ensure effectiveness.

**Figure 3:**
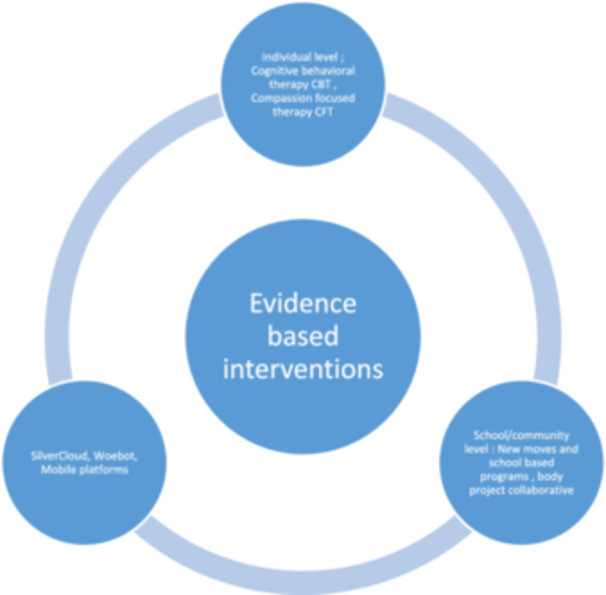
Evidence‐based interventions for body shaming across levels of care. The figure illustrates three interconnected levels of evidence‐based interventions targeting body shaming and body image disturbance. At the individual level, psychological therapies including Cognitive Behavioral Therapy (CBT) and Compassion‐Focused Therapy (CFT) are employed to address negative self‐perception and internalized stigma. At the school and community level, structured prevention programs such as the Body Project Collaborative and New Moves provide group‐based approaches to reduce body dissatisfaction among young people. Digital and mobile platforms, including SilverCloud and Woebot, represent emerging technology‐driven solutions that expand access to mental health support beyond traditional clinical settings. The overlapping design reflects the complementary and mutually reinforcing nature of these interventions in addressing body shaming comprehensively.

Collectively, these strategies indicate that structured, psychologically grounded interventions, whether delivered through therapy, schools, or digital platforms, can reduce internalized stigma and mitigate the mental health burden of body shaming provided they are carefully adapted to the realities of LMIC settings. Figure 3 demonstrates in summary the evidence‐based interventions.

### 2.4 Emerging Awareness and Initiatives in African Contexts

Although research on body shaming in Africa remains limited, recognition of the issue is growing within public health and gender equity discussions. Traditionally, many African cultures have celebrated fuller body shapes, associating them with health, fertility, and prosperity [28]. However, Western beauty norms, often propagated through media, urbanization, and social media platforms, are increasingly influencing local perceptions, contributing to rising body dissatisfaction and the emergence of eating disorders in urban centers [33, 34]. For instance, a study in Nigeria highlights a paradoxical dynamic: while cultural practices promote weight gain as desirable, young women experience heightened anxiety and self‐consciousness due to globalized beauty ideals [17].

Despite this emerging awareness, there is a notable absence of large‐scale, empirical studies across African settings, particularly those examining rural populations, intersectional factors such as age, socioeconomic status, and disability, or longitudinal trends. This scarcity of context‐specific evidence represents a critical research gap that limits the development of culturally sensitive policies and interventions. Nevertheless, local NGOs and youth‐led campaigns are beginning to address these issues in culturally resonant ways. Additionally, the African Union's Agenda 2063 emphasizes the integration of mental health, gender equity, and cultural resilience into policy, offering a framework to support interventions targeting body image and related psychosocial outcomes [35].

This awareness lays the groundwork for culturally relevant, evidence‐based interventions in Africa. For example, initiatives such as the Dove Self‐Esteem Project in South Africa have promoted body positivity among adolescents through school workshops and media campaigns [13, 16]. In Nigeria, pilot school‐based programs integrating media literacy and self‐esteem education have addressed harmful beauty norms among urban youth [17]. Additionally, task‐shifting mental health interventions in Mozambique and South Africa, delivered by trained lay health workers, have incorporated psychoeducation on self‐esteem and body image into broader psychosocial support services [25, 27]. These examples demonstrate that culturally adapted interventions, ranging from school programs to community and digital initiatives, can be feasible in African settings and provide important lessons for scaling up evidence‐based strategies.

Table 1 summarizes selected evidence on body shaming and its mental health implications within African contexts, highlighting both emerging findings and significant research gaps.

**Table 1:** Evidence on body shaming and mental health in selected African contexts.

Country/region	Study/intervention focus	Population	Key findings	Public health implications
Nigeria	Cultural norms and globalized beauty ideals [17]	Young women	Paradoxical dynamic: traditional norms promote weight gain as desirable, yet exposure to Western beauty standards increases anxiety and self‐consciousness	Need for culturally adapted mental health and media literacy interventions targeting young women
Urban African Settings (Nigeria, South Africa)	Media influence and body dissatisfaction [3]	Urban adolescents and young women	Increasing exposure to Western beauty norms associated with rising body dissatisfaction and emerging eating disorders in urban centers	Highlights urgency of early screening and school‐based prevention programs
Multiple African Contexts	Cultural body ideals [25]	Women across various communities	Fuller body shapes traditionally associated with health, fertility, and prosperity	Interventions must balance respect for cultural values with promotion of psychological wellbeing
South Africa	Dove Self‐Esteem Project [13]	Adolescents and young women	Social media and school campaigns improved self‐esteem and body confidence	Demonstrates potential for digital and school‐based campaigns to shift body image perceptions
Kenya	Youth‐led NGO campaign “Love Your Shape” [36]	Urban and peri‐urban youth	Peer‐led workshops promoted body positivity, reducing reported stigma and self‐criticism	Highlights value of youth‐led, culturally relevant interventions for scalable behavior change
Ghana	Community awareness and media literacy program [12]	Adolescent girls	Combination of school‐based education and radio messaging reduced internalized thin‐ideal perceptions	Suggests multiplatform interventions can increase reach and impact across LMIC contexts
Continental Policy Level (African Union)	Agenda 2063 framework [35]	Policy‐level	Emphasis on integrating mental health, gender equity, and cultural resilience into development strategies	Provides policy framework to embed body image and mental health within broader gender equity agendas

## 3 Challenges in Addressing Body Shaming

**Figure 4:**
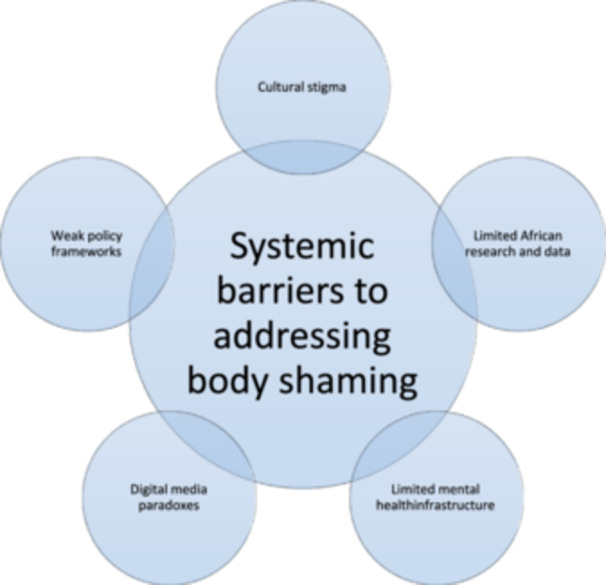
Systemic barriers to addressing body shaming in women. The diagram illustrates the interconnected structural, cultural, digital, health system, and policy‐level challenges that collectively hinder effective prevention and response efforts.

Despite growing recognition and emerging interventions, several challenges continue to limit progress in combating body shaming among women. Figure 4 illustrates the interconnected systemic barriers that limit progress in addressing body shaming among women, highlighting the multilevel nature of the challenge.

### 3.1 Cultural Stigma

Deeply entrenched beauty standards reinforced by media, family, and peer groups remain a major barrier. Cultural ideals vary widely, and attempts to promote body positivity can sometimes conflict with local values or provoke resistance, particularly in societies where certain body types are equated with health, fertility, or social status [1, 3, 4, 12, 17]. These societal pressures can undermine the uptake of mental health interventions and hinder the promotion of diverse body representations [2, 5].

### 3.2 Limited Research and Data in African Contexts

Compared to Western settings, research on body shaming and its psychosocial impact in Africa is sparse. Small sample sizes, urban‐centered studies, and a lack of longitudinal data restrict understanding of prevalence, risk factors, and effective interventions [2, 17, 28, 36]. This scarcity of context‐specific evidence limits the development of culturally appropriate policies and programs.

### 3.3 Limited Mental Health Infrastructure

Access to mental health services remains uneven, particularly in low‐ and middle‐income countries. Limited availability of trained psychologists, stigma around seeking mental health support, and lack of school‐based or community interventions impede early detection and management of body image‐related distress [25, 27, 32]. Consequently, many women suffer in silence, exacerbating the long‐term consequences of body shaming.

### 3.4 Digital Media Paradoxes

While social media can foster body positivity campaigns, it simultaneously reinforces harmful beauty ideals through curated images, filters, and algorithmic driven content [2, 3, 22]. Balancing these opposing effects presents a complex challenge for policymakers, educators, and mental health practitioners.

### 3.5 Weak Policy Frameworks

Although initiatives like the African Union's Agenda 2063 recognize the importance of mental health and gender equity, concrete policies addressing body shaming remain scarce. Limited enforcement, insufficient funding, and absence of cross‐sectoral collaboration hinder the translation of policy into meaningful community‐level interventions as well as reduce the effectiveness of existing initiatives [17, 35].

These challenges collectively illustrate that addressing body shaming requires multilevel strategies, combining culturally sensitive research, expanded mental health services, digital literacy programs, and strong policy frameworks.

## 4 Future Directions

Addressing body shaming as a public health and gender equity priority requires a shift from fragmented, reactive responses toward integrated, proactive frameworks. Current evidence demonstrates the psychological, social, and health consequences of appearance‐based stigma, yet interventions across clinical, educational, digital, and policy domains remain poorly coordinated. This section proposes five interconnected future directions to bridge that gap. Table 2 summarizes these directions according to urgency, feasibility, and expected public health impact, providing a strategic basis for prioritization.

**Table 2:** Priority roadmap for addressing body shaming as a public health and gender equity issue.

Priority rank	Strategic area	Key actions	Urgency	Feasibility in LMICs	Expected public health impact
1	Research Intersectional and culturally sensitive research	Conduct longitudinal and context‐specific studies; develop culturally validated measurement tools; include marginalized populations (rural women, women with disabilities, low‐income groups).	High	Moderate (requires funding but scalable through academic partnerships)	High—Builds foundational evidence for policy and intervention design
2	Healthcare Integration Mental health and primary care	Introduce routine screening for body image distress; adapt CBT, CFT, and school‐based programs; train primary care providers in brief psychosocial interventions.	High	Moderate (requires workforce training and task‐shifting approaches)	High—Early identification reduces long‐term psychological burden
3	Education and digital literacy	Integrate media literacy into school curricula; implement youth resilience programs; collaborate with digital platforms for content moderation and body‐positive campaigns.	Moderate–High	High (school‐based and digital models are scalable)	Moderate–High—Preventive impact across large youth populations
4	Policy and multisectoral action	Develop national frameworks integrating body image into gender and mental health strategies; allocate funding; align with SDG3 and SDG5 targets.	Moderate	Moderate (dependent on political commitment)	High—Enables sustainable systemic change
5	Empowerment and community engagement	Support peer‐led initiatives; promote community dialogs; amplify women's voices; implement culturally grounded awareness campaigns.	Moderate	High (community‐based models are cost‐effective)	Moderate–High—Shifts social norms and reduces stigma

### 4.1 Research: Intersectional and Culturally Sensitive Research

Future studies must adopt intersectional approaches, examining how age, culture, socioeconomic status, disability, and urban–rural contexts shape experiences of body shaming among women in Africa. Current evidence is heavily inclined toward urban, educated, and relatively affluent populations, leaving rural women, women with disabilities, older women, and those in low‐income households largely absent from the research base, a gap that directly limits the relevance of existing intervention models for the majority of African women [2, 28]. In Africa specifically, research should prioritize the development and validation of culturally relevant measurement tools, given that widely used instruments such as the Body Shape Questionnaire (BSQ) and the Eating Attitudes Test were developed and normed in Western populations and may not adequately capture the culturally specific expressions of body dissatisfaction and appearance‐based stigma present in African contexts [17, 36]. Longitudinal designs are equally critical—cross‐sectional studies dominate the existing literature and cannot establish whether body shaming precedes mental health deterioration or whether pre‐existing psychological vulnerability amplifies susceptibility to appearance‐based stigma. Community‐based participatory research methods, which position affected women as co‐investigators rather than research subjects, offer a particularly promising approach for generating findings that are both contextually grounded and directly actionable at the community level [23]. Understanding these intersectional nuances is not merely an academic exercise, it is the foundational condition for designing interventions that resonate with local values, earn community trust, and leverage global best practices without reproducing the cultural blind spots that have constrained progress to date.

### 4.2 Healthcare Integration: Mental Health and Primary Health Care

Screening for body image‐related distress should become routine in primary care, reproductive health services, and mental health clinics, particularly for adolescents and young women. Simple, validated screening tools such as the BSQ and the Eating Attitudes Test (EAT‐26) are brief enough for integration into existing consultation workflows without requiring specialist training, making them feasible for use in primary care settings across African health systems [18, 21]. Early identification through these pathways is essential, body image‐related distress that goes undetected at the primary care level frequently escalates into clinical depression, disordered eating, and sustained social withdrawal before any intervention is initiated. Evidence‐based interventions, including CBT, CFT, and school‐based programs such as the Body Project, must be adapted and scaled within healthcare systems through task‐shifting models that deploy trained community health workers, teachers, and peer educators rather than relying on specialist psychologists whose availability across sub‐Saharan Africa remains critically limited [25, 27]. Scaling these models requires investment in training curricula, supervision infrastructure, and fidelity monitoring; conditions that must be built into program design from the outset rather than treated as implementation afterthoughts. Together, routine screening and task‐shifted intervention delivery represent the most realistic pathway to early detection and reduction of the long‐term psychological and social consequences of body shaming in resource‐constrained settings.

### 4.3 Education and Digital Literacy

Incorporating media literacy and body positivity curricula in schools can build resilience early, equipping young people to critically interrogate the idealized and digitally altered images they encounter daily on social media platforms. Evidence from school‐based programs in Nigeria and South Africa demonstrates that embedding media literacy within existing health education classes rather than introducing standalone curricula produces stronger uptake and more durable attitudinal change among adolescents [17, 32]. Parallel efforts must engage digital platforms directly, encouraging algorithmic transparency, content moderation standards, and partnerships with body‐positive influencers to shift online norms at scale. Programs such as Dove's #DetoxYourFeed and TikTok's body‐positive content guidelines, which ban harmful diet‐related material and promote diverse body representation, demonstrate that digital environments can be intentionally structured to support rather than undermine mental wellbeing [13, 16]. Importantly, these digital and school‐based strategies are most effective when deployed as upstream prevention, targeting younger populations before internalized body shame consolidates into clinical levels of distress. In low‐resource African settings, radio broadcasting and community messaging platforms offer complementary channels for reaching girls and young women in rural areas where smartphone access and internet connectivity remain limited, extending the reach of digital literacy efforts beyond urban centers [12].

### 4.4 Policy and Multisector Action

Addressing body shaming requires cross‐sector collaboration between healthcare providers, educators, media regulators, NGOs, and policymakers, with dedicated funding for research, community programs, and public awareness campaigns. Policy frameworks inspired by the African Union's Agenda 2063 should explicitly integrate body image and mental health goals into gender equity and development agendas. Beyond broad frameworks, however, effective policy must include enforceable regulatory measures targeting social media platforms, where algorithmic amplification of appearance‐based content causes demonstrable psychological harm to women and girls. Regional bodies such as the East African Community and the Southern African Development Community are well‐positioned to develop shared digital content standards across member states. At the country level, Kenya's Computer Misuse and Cybercrimes Act (2018) provides a legislative foundation that can be extended to cover algorithmic promotion of appearance‐shaming material [37]. South Africa's Films and Publications Amendment Act (2019) establishes precedent for regulating harmful digital content, including body‐shaming material in commercial advertising [38]. Nigeria's National Information Technology Development Agency holds an existing mandate to issue platform conduct guidelines covering appearance‐based cyberbullying, having previously engaged social media companies on harmful content standards [39]. Across these contexts, platform obligations should include algorithmic opt‐out mechanisms for appearance‐comparison content, mandatory disclosure of digitally altered images in advertising, and accessible reporting channels for appearance‐based harassment measures that move beyond the voluntary intent of TikTok's body‐positive content guidelines toward enforceable national obligations.

### 4.5 Empowerment and Community Engagement

Finally, solutions must center women's voices, leveraging community‐driven initiatives and peer‐led programs to foster self‐acceptance, social support, and empowerment. Women are not merely beneficiaries of interventions addressing body shaming, they are the most credible agents of norm change within their own communities, families, and peer networks. Peer‐led programs that train young women as body image advocates within schools and community settings have demonstrated stronger uptake and sustained behavior change than externally facilitated programs, precisely because they operate within existing social trust structures [23, 36]. Culturally grounded campaigns that draw on local languages, storytelling traditions, and community gatherings can shift societal norms, reduce stigma, and create environments in which diverse body types are celebrated rather than penalized. Engaging religious and community leaders in these efforts is particularly important in African contexts, where such figures carry significant normative influence over how women's bodies are perceived and discussed. Ultimately, empowerment and community engagement are not supplementary to clinical and policy action, they are the condition under which such action becomes socially legitimate and therefore sustainable.

## 5 Conclusion

Tackling body shaming is not merely about promoting individual resilience rather it requires systemic change across healthcare, education, media, and policy spheres. Coordinated global and local efforts, informed by evidence and driven by cultural sensitivity, can help women achieve healthier self‐perceptions, enhanced mental wellbeing, and true social equity. Addressing body shaming must be embedded into African public health and gender equity frameworks to ensure measurable progress on SDG3 and SDG5.

## 6 Limitations

Despite the comprehensive review and synthesis presented, this article has several limitations. First, there is a notable publication bias, as most available studies originate from high‐income countries, limiting generalizability to African contexts. Second, data from sub‐Saharan Africa remain sparse, with few large‐scale or longitudinal studies exploring body shaming and its mental health impacts among women. Third, the reliance on secondary sources and literature reviews may overlook unpublished interventions or local community‐driven initiatives. Finally, cultural diversity across African societies means that findings from one setting may not fully reflect experiences in others, highlighting the need for context‐specific research. Recognizing these limitations underscores the urgency of generating robust, locally relevant evidence to inform policies and interventions targeting body shaming among women in Africa.

## Author Contributions

**Shani Kondo Omari:** conceptualization, literature review, writing – original draft, supervision. **Lydia Issaria Mosses:** literature review, editing. **Hussein Hassan Mwanjali:** literature review, editing. All authors have read and approved the final version of the manuscript.

## Funding

The authors have nothing to report.

## Ethics Statement

The authors have nothing to report.

## Conflicts of Interest

The authors declare no conflicts of interest.

## Transparency Statement

Shani Kondo Omari affirms that this manuscript is an honest, accurate, and transparent account of the study being reported; that no important aspects of the study have been omitted; and that any discrepancies from the study as planned (and, if relevant, registered) have been explained.

## Data Availability

No new data were generated or analyzed in support of this manuscript. The corresponding author takes complete responsibility for the accuracy and integrity of the manuscript content. No new data analysis was conducted for this perspective article.
